# Biomarker evidence of early vision and rod energy-linked pathophysiology benefits from very low dose DMSO in 5xFAD mice

**DOI:** 10.1186/s40478-024-01799-8

**Published:** 2024-05-31

**Authors:** Bruce A. Berkowitz, Anuhya Paruchuri, Josh Stanek, Mura Abdul-Nabi, Robert H. Podolsky, Abner Heredia Bustos, Karen Lins Childers, Geoffrey G. Murphy, Katherine Stangis, Robin Roberts

**Affiliations:** 1 Department of Ophthalmology, Visual and Anatomical Sciences, Wayne State University School of Medicine, 540 E. Canfield, Detroit, MI 48201 USA; 2 Biostatistics and Study Methodology, Children’s National Hospital, Silver Spring, MD USA; 3 CSCAR, University of Michigan, Ann Arbor, MI USA; 4 Beaumont Research Institute, Beaumont Health, Royal Oak, MI 48073 USA; 5 Department of Molecular and Integrative Physiology, Molecular Behavioral Neuroscience Institute, University of Michigan Medical School, Ann Arbor, MI USA; 6 Michigan Neuroscience Institute, University of Michigan Medical School, Ann Arbor, MI USA

**Keywords:** Photoreceptor, OCT, Biomarker, Mitochondria, Energy, Acidification

## Abstract

Here, we test whether early visual and OCT rod energy-linked biomarkers indicating pathophysiology in nicotinamide nucleotide transhydrogenase (*Nnt*)-null 5xFAD mice also occur in *Nnt-*intact 5xFAD mice and whether these biomarkers can be pharmacologically treated. Four-month-old wild-type or 5xFAD C57BL/6 substrains with either a null (B6J) *Nnt* or intact *Nnt* gene (B6NTac) and 5xFAD B6J mice treated for one month with either R-carvedilol + vehicle or only vehicle (0.01% DMSO) were studied. The contrast sensitivity (CS), external limiting membrane-retinal pigment epithelium (ELM-RPE) thickness (a proxy for low pH-triggered water removal), profile shape of the hyperreflective band just posterior to the ELM (i.e., the mitochondrial configuration within photoreceptors per aspect ratio [MCP/AR]), and retinal laminar thickness were measured. Both wild-type substrains showed similar visual performance indices and dark-evoked ELM-RPE contraction. The lack of a light–dark change in B6NTac MCP/AR, unlike in B6J mice, is consistent with relatively greater mitochondrial efficiency. 5xFAD B6J mice, but not 5xFAD B6NTac mice, showed lower-than-WT CS. Light-adapted 5xFAD substrains both showed abnormal ELM-RPE contraction and greater-than-WT MCP/AR contraction. The inner retina and superior outer retina were thinner. Treating 5xFAD B6J mice with R-carvedilol + DMSO or DMSO alone corrected CS and ELM-RPE contraction but not supernormal MCP/AR contraction or laminar thinning. These results provide biomarker evidence for prodromal photoreceptor mitochondrial dysfunction/oxidative stress/oxidative damage, which is unrelated to visual performance, as well as the presence of the *Nnt* gene. This pathophysiology is druggable in 5xFAD mice.

## Introduction

Mild cognitive impairment is an early condition in patients who are often subsequently diagnosed as having Alzheimer’s disease (AD). Similarly, experimental models (e.g., the 5xFAD mouse) that mimic hallmark AD neuropathology exhibit cognitive deficits [1, 2]. Since its development in 2006, the 5xFAD mouse, which overexpresses human *APP* with three FAD mutations [the Swedish (K670N, M671L), Florida (I716V), and London (V7171) mutations] and human *PSEN1* with two FAD mutations (M146L and L286V), has been used in 1,423 studies to date (PubMed search 04/23/24) [1]. One promising area for therapeutic intervention is the link between neuronal mitochondrial dysfunction (e.g., hyperactivity), oxidative stress (i.e., an imbalance between free radical production and suppression), and damage produced by prolonged oxidative stress to, for example, mitochondria. In studies often performed ex vivo, these interlinked abnormalities have been identified before the appearance of overt pathology and degeneration (i.e., prodromally) in AD [2–25]. New translational imaging biomarkers are needed to evaluate treatment efficacy early in the course of the disease in animal models and in patients.

Intriguingly, the retina, which is embryologically derived from brain tissue, shows characteristic AD pathology (i.e., soluble amyloid β-peptide oligomers and plaque, phosphorylated tau and neurofibrillary tangles) well before the appearance of AD pathology in the brain [16–21, 26, 27]. In addition, there is evidence for retinal oxidative stress in a common experimental model of AD, 5xFAD mice, as early as 1 month of age [28–30]. For example, preventative treatment with an anti-oxidant, methylene blue, can mitigate cognitive declines in experimental AD [29, 30]. Also, during the initial stages of AD, visual performance (e.g., contrast sensitivity) declines, and there appears to be prodromal rod mitochondrial dysfunction as measured by biomarkers obtained from readily accessible and relatively inexpensive optical coherence tomography (OCT) in patients with mild cognitive impairment and in animal AD models [24, 31–38] .

Imaging biomarkers can also be used to evaluate outer retinal oxidative stress [38, 39]. For example, in light-adapted mice, outer retinal oxidative stress elicited by the administration of systemic diltiazem, a calcium channel blocker, or streptozotocin-induced diabetes, and confirmed by gold-standard assays, were associated with a contracted external limiting membrane—retinal pigment epithelium (ELM-RPE) measured from OCT or diffusion MRI [38, 39]. The administration of anti-oxidants corrected the ELM-RPE contraction [38, 39]. These results are in line with the presence of oxidative stress-induced acidification of the subretinal space, and ELM-RPE thickness being a downstream index for pH-triggered / RPE-water removal from the subretinal space [39–46]. At this time, there is inconclusive evidence to link visual performance declines and the OCT ELM-RPE rod mitochondrial impairment / oxidative stress biomarker. In this study, visual performance and ELM-RPE indices are considered to be separate manifestations of early AD. Regardless, further OCT studies of the retina appear warranted as a rational and readily available approach for early diagnosis, and / or testing the bioactivity of novel treatments with the potential to modify the trajectory of AD.

Experimentally, evidence for visual performance declines and an apparent rod mitochondria dysfunction / hyperactivity have been obtained from commercially-available 5xFAD and 3xFAD mouse models on a wildtype (WT) substrain background null for the nicotinamide nucleotide transhydrogenase (*Nnt*) gene (i.e., C57BL/6J background). In the absence of *Nnt*, there is a lower-than-normal production of nicotinamide adenine dinucleotide phosphate (NADPH) which is an essential catalyst for many enzymes that detoxify reactive oxygen species [47–49]. Subnormal production of NADPH can thus increase the level of reactive oxygen species which in turn lowers how efficiently mitochondria generate ATP per unit of consumed oxygen via oxidative phosphorylation (i.e., the energy released involved in the production of ATP via movement of electrons to oxygen) [47–49]. However, it is unclear if the absence of *Nnt* contributes to the pattern of change in energy-linked biomarkers and thus confound translational efforts since this mutation is not typically found in patients at risk for AD. New evidence suggests that rod mitochondria dysfunction, but perhaps not rod hyperactivity per se could explain the reported changes in OCT biomarkers in 5xFAD mice.

We have identified another OCT energy-linked biomarker in photoreceptors that has also been validated against gold-standard assays [39, 43–45, 50–52]. This is the Mitochondria Configuration within Photoreceptors (MCP), measured by its Aspect Ratio (AR) of the hyperreflective band immediately posterior to the ELM [53–55]. As a proxy for the spatial distribution and number of mitochondria within the ellipsoid of the photoreceptors, MCP/AR appears to be an important metric of photoreceptor cell functioning and survival [53–55]. Based on these considerations, MCP/AR is considered an index of the respiratory efficiency of the photoreceptor [56, 57].

Both ELM-RPE thickness and MCP/AR are sensitive to physiologic differences in photoreceptor energy production in WT mice with intact (129S6/ev) or null (B6J) *Nnt* [47, 50–52, 56, 58]. Both mouse strains show contraction of the ELM-RPE region under high energy demand (dark adaptation) vs. that in the light but only B6J mice show MCP/AR to be greater in the dark than in the light, an indication of rod mitochondria inefficiency [50–52, 56, 58]. It is unclear if other inbred mouse strains, such as B6 mice with intact *Nnt*, show a similar pattern in ELM-RPE thickness and MCP/AR values in light–dark as those measured in 129S6/ev mice, as well as the OCT biomarker pattern suggesting mitochondria dysfunction / oxidative stress (i.e., contracted ELM-RPE, supernormal MCP/AR) as measured in 5xFAD B6J mice [24, 56].

Strikingly, it is possible to pharmacologically restore cognitive function and suppress neuronal hyperactivity without altering plaque deposition in mouse models of amyloidogenesis [7, 26, 59–72]. For example, treatment with R-carvedilol—a drug that limits the open-time of the endoplasmic reticulum (ER) ryanodine receptor type 2 (RyR2) calcium channel—corrects both neuronal hyperactivity and the trajectory of cognitive loss in these models [7, 26, 59–63, 65, 67, 68, 72–74]. It is not clear if the R-carvedilol enantiomer has anti-oxidant properties. The impact of R-carvedilol—or its vehicle DMSO—on visual performance declines and rod OCT biomarkers in 5xFAD mice remains unclear [24].

In this study, we test three hypotheses: (1) that *Nnt-*intact WT B6 mice show OCT biomarker evidence for greater mitochondrial efficacy than *Nnt*-null WT mice (B6J), (2) that prodromal visual performance is impaired and OCT rod biomarkers show evidence for mitochondria dysfunction in *Nnt-*intact 5xFAD mice, and (3) that R-carvedilol or its vehicle DMSO given to 5xFAD B6J mice improve the early impairments in contrast sensitivity and abnormal rod OCT biomarker patterns.

## Methods

All mice were treated in accordance with the National Institutes of Health Guide for the Care and Use of Laboratory Animals, the Association for Research in Vision and Ophthalmology Statement for the Use of Animals in Ophthalmic and Vision Research, and with specific authorization by the Wayne State University Division of Laboratory Animal Resources Institutional Animal and Care Use Committee (IACUC). We studied the following groups: 4 mo male non-littermate WT C57BL/6NTac (B6NTac; Taconic Laboratories, Germantown, NY, USA), 5xFAD mice B6NTac (5xFAD B6NTac, Dr. Geoffrey Murphy), 4 mo non-littermate WT C57BL/6J mice (B6J; Jackson Laboratories, Bar Harbor, ME, USA), and 5xFAD mice C57BL/6J mice (5xFAD B6J, #000664 and #034848, respectively; The Jackson Laboratory), 5xFAD B6J mice treated with R-carvedilol (Musechemicals) + 0.01% DMSO (Sigma-Aldrich) orally (added to drinking water) from 3 to 4 mo of age, and B6J mice treated with 0.01% DMSO in the water from 3 to 4 mo of age. R-carvedilol (13.2 mg dissolved in 100 μl DMSO) was diluted 10, 000 with distilled water and added to the drinking water so that mice consumed a dose of ~ 3.2 mg/kg/day based on the assumed water consumption of similarly sized mice, the protocol of Yao et al. [62]. Preliminary studies found that both R-carvedilol and DMSO appeared stable (i.e., no degeneration products) after one month of room temperature and light exposure based on unremarkable liquid chromatography with simultaneous mass spectrometry (LC–MS) and UV (phot diode array) detection (LC-PDA) measurements (Shimadzu Nexera X2 UPLC PDA-8040 MS system) (data not shown).

All mice were housed and maintained in 12-h/12-h light–dark cycle laboratory lighting. After scanning, mice were humanely euthanized by an overdose of ketamine/xylazine followed by cervical dislocation. All procedures were approved by the Wayne State University Institutional Animal and Care Use Committee. Data were collected from the left eye.

### Optokinetic tracking (OKT)

Two visual performance metrics were measured from awake and freely moving mice. The first is the spatial frequency threshold (“acuity,” in cyc/deg (c/d)), or the highest grating spatial frequency that triggered head tracking [75, 76]. Contrast sensitivity was measured taking the inverse spatial frequency threshold (i.e., the inverse Michelson contrast (unitless)) measured at a grating setting of 0.06 c/d (near the nominal peak contrast sensitivity) using the optokinetic tracking (OKT) reflex (OptoMotry; CerebralMechanics, Inc., Lethbridge, AB, Canada), as described previously [75, 76]. In brief, a vertical sine wave grating is projected as a virtual cylinder in three-dimensional coordinate space on computer monitors arranged in a quadrangle around a testing arena. Unrestrained mice were placed on an elevated platform at the center of the arena. An experimenter used a video image of the arena from above to view the animal and follow the position of its head with the aid of a computer mouse and a crosshair superimposed on the mouse head. The *x*, *y* positional coordinates of the crosshair are centered on the hub of the virtual cylinder, enabling its wall to be maintained at a constant “distance” from the animal's eyes and thereby adjusting the spatial frequency of the stimulus to a fixed viewing position. When the cylinder was rotated in the clockwise (CW) or counterclockwise (CCW) direction and the animal followed with head and neck movements that tracked the rotation, it was judged whether the animal's visual system could distinguish the grating. CW and CCW tracking provide a measure of left and right eye acuity and contrast sensitivity [75, 77]. One set of acuity and peak of contrast sensitivity measurements can reliably be obtained in 30 min.

### Optical coherence tomography (OCT)

In a separate cohort of mice, a cross-sectional design was used herein in which anesthetized mice—100 mg/kg ketamine (Covetrus, Portland, ME, USA) and 6 mg/kg xylazine (MWI Animal Health, Boise, ID, USA)—were examined by OCT (Envisu UHR2200; Bioptigen, Durham, NC, USA) in the morning (i.e., before noon). WT mice were studied after either dark adaption overnight followed by 1 h of room light prior to imaging or kept in the dark in different groups of mice on different days; we find these conditions detect light and dark changes in ELM-RPE thickness and MCP/AR without inducing handling stress from multiple rounds of anesthesia. The 5xFAD mice were dark adapted overnight, and the following day, room-light adapted for just 1 h room light prior to imaging. In all OCT studies, the iris was dilated with 1% atropine sulfate, and Systane Ultra (Alcon, Geneva, Switzerland) was used to lubricate the eyes.

From central retina, we collected radial volume scans with the following parameters: A-scans/B-scans = 1000 lines; B-scans/volume = 1000 scans; and frames/B-scan = 1 frame. One hundred images extracted from B-scan numbers 450 to 549 (representing inferior–superior retina) were registered (in-house script for R; R Foundation for Statistical Computing, Vienna, Austria). Briefly, first-pass rigid body registration with RNiftyReg (function in R) was used to rotate the image and interpolate the signal at each pixel. Next, non-rotational rigid-body approaches (at the level of a given row or column of pixels) were applied three times. The 100 images were visually compared as a final step before averaging.

### Image analysis

Laminar boundaries for segmentation were estimated with a previously described machine learning model–based computer program [46]. Briefly, the machine learning model was a U-net convolutional neural network trained using the “dice loss” function and the Adam optimizer (learning rate = 0.001), with 665 previously labeled images for training and 166 and 356 images for validation and testing, respectively. To improve the performance of the model, its predictions were postprocessed by applying a shortest-path algorithm. From the model-based estimates, segmentation was then performed with an R script. The intensity values used to generate the EZ reflectivity profile shape are measured from a log-based image with a 16-bit depth (default in the Bioptigen system). Previously, we compared images before and after converting them from log to linear values using a simple, empirically derived equation and noted that EZ reflectivity profile shape differences would be detected with either output; more detailed work in this area is warranted.

Once segmented, inferior and superior retinas (350–624 µm from the optic nerve head on the inferior and superior sides) were each analyzed; starting at 350 µm ensured that our data were analyzed away from the optic nerve head, where the outer retina is relatively uniform in all OCT data. We measured the ELM-RPE thickness using in-house R scripts that objectively extracted layer boundaries obtained after searching the space provided by a machine-learning estimates (“seed boundaries”) as above. The ELM and RPE are initially identified by local signal maxima and the R script determine the ELM-RPE thickness by calculating the distance from ELM to the basal side of the RPE at the level of Bruch’s membrane [78–80].

Our analysis generated a spreadsheet of distances from the optic nerve head and layer thickness. The output image had the basal aspect of the RPE held in a fixed position without stretching the image; this non-stretched image was used to generate the A-line reflectivity profiles shown herein.

We analyzed MCP/AR using an unbiased approach in which MATLAB code (MathWorks, Natick, MA, USA) determines the baseline for hyperreflective band immediately posterior to the ELM. The profile shape descriptor for this reflectivity profile was generated using the Fit Ellipse command in ImageJ. In the results window, the value under the column marked “round” is the minor-to-major aspect ratio for the fitted ellipse, termed herein as MCP/AR; this process is described at https://imagej.nih.gov/ij/source/ij/process/EllipseFitter.java and more formally in the literature [81]. It remains to be determined if the ellipse aspect ratio, a commonly used shape descriptor; is an optimal shape description for these types of studies, nonetheless it remains useful [24, 56, 57]. Also, our experience to date is that MCP/AR is not a function of ELM-RPE thickness, an impression supported by the data in this report (see below).

Previously, we had investigated another OCT energy-sensitive biomarker, the hyporeflective band between the photoreceptor tips and apical RPE. However, since we have found that the hyporeflective band does not appear to provide additional information over that provided by the better understood ELM-RPE thickness, it is not reported herein.

### Statistical analyses

Data are presented as mean and 95% confidence intervals, and we used *p* < 0.05 to indicate statistically significant differences for all analyses. We used generalized linear mixed models with the Kenward-Roger method for calculating degrees of freedom in SAS 9.4 (SAS software, Cary, NC, USA) to analyze all measurements. We first averaged all values for all variables across depth/distance separately for each side (inferior/superior). All models included a random intercept for mouse within group (strain or treatment group) and condition (light/dark) where appropriate. For all models, we initially included all interactions among fixed effects, removing non-significant higher-order interactions to arrive at a final model. Linear contrasts were used as needed to evaluate only effects that were significant in the model. We did not adjust for multiple testing since all comparisons were planned, and we never did all pairwise comparisons. We used an identity link and normal distribution for all measures except for contrast sensitivity (CS) for which we used a log link and the gamma distribution. These links and distributions were chosen based on the distribution of residuals from the generalized linear mixed model.

We analyzed B6J and B6NTac wild-type mice together to evaluate differences in these backgrounds. To analyze CS and acuity, we included the fixed effects of direction (clockwise [CW], counterclockwise [CCW]) and strain (B6J, B6NTac). To analyze MCP/AR and laminar layer thickness (e.g., ELM-RPE thickness), we included the fixed effects of strain, side (inferior vs superior), and condition.

The 5xFAD mutation was compared to WT separately for both the B6J and B6NTac backgrounds. The models used for these backgrounds differed since DMSO and R-carvedilol were only administered to B6J mice. For B6NTac mice, we included the fixed effects of direction and strain (WT, 5xFAD) for CS and acuity, and side and strain for MCP/AR and laminar layer thicknesses. For B6J mice, we included the fixed effects of direction (acuity and CS) or side (MCP/AR and laminar layer thickness), and group (WT untreated, 5xFAD untreated, 5xFAD DMSO, 5xFAD DMSO + R-carvedilol).

## Results

### Hypothesis 1: WT substrains

Our previous studies in WT mice suggested that visual performance was similar—and OCT energy biomarkers were different—between mice strains which either did have (129S6ev) or did not have (B6J) *Nnt* [56, 82]. Thus, we tested the hypothesis that the WT B6NTac mouse would show different patterns in these indices than in the B6J mouse.

#### OKT

**Fig. 1:**
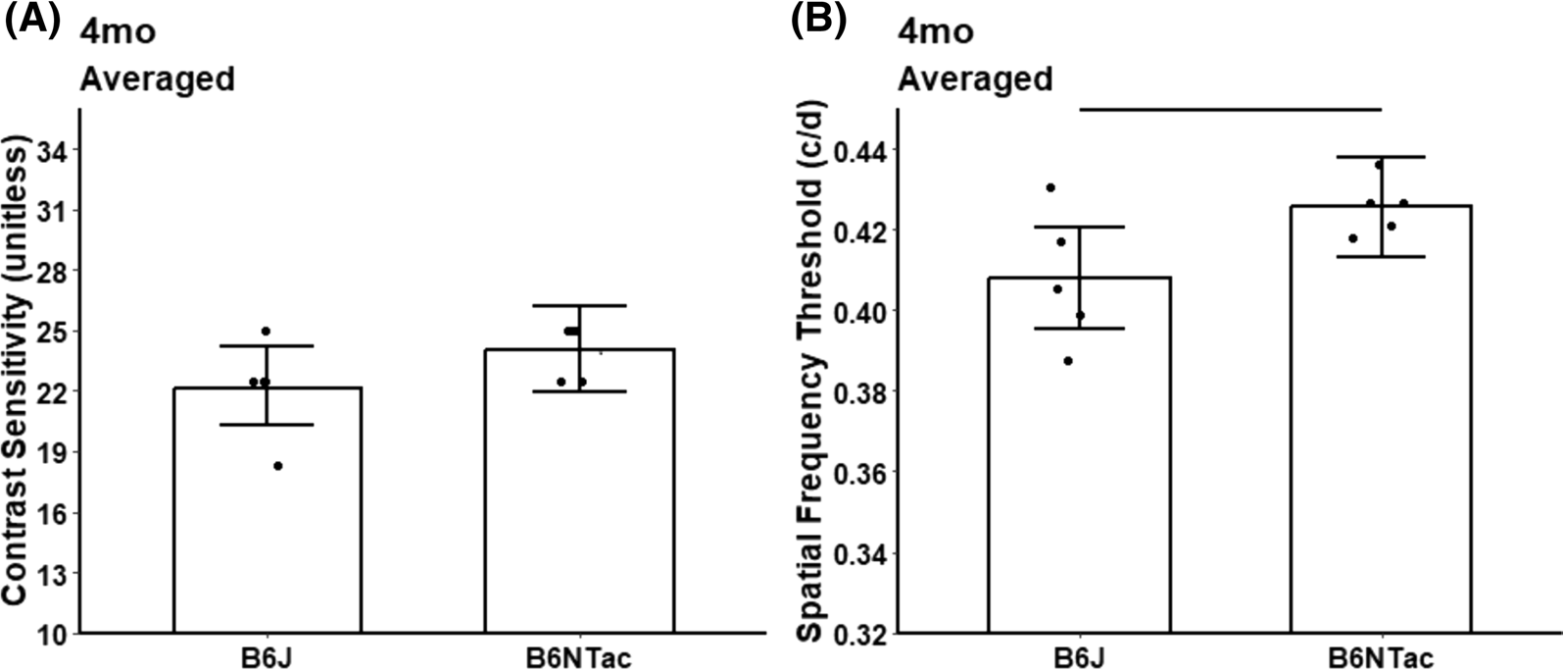
Summary of visual performance in WT substrains. No evidence for different contrast sensitivity **A** was noted although differences in acuity **B** were shown; n = 5 B6J, n = 5 B6NTac. Individual data points represent the measured value for each mouse. Potential strain differences in both indices did not depend on which direction the grating moved, and means did not differ between directions so averages over both directions are presented

No significant difference in CS was found between 4 mo B6J and B6NTac mice (fold change = 0.9, 95% CI 0.8–1.1, *p* = 0.19) implying an independence of visual performance from *Nnt* (Fig. 1). However, 4 mo B6NTac mice had greater acuity than B6J mice (mean difference = 0.02 c/d, 95% CI 0.0001–0.04, *p* = 0.05).

#### OCT energy biomarkers

**Fig. 2:**
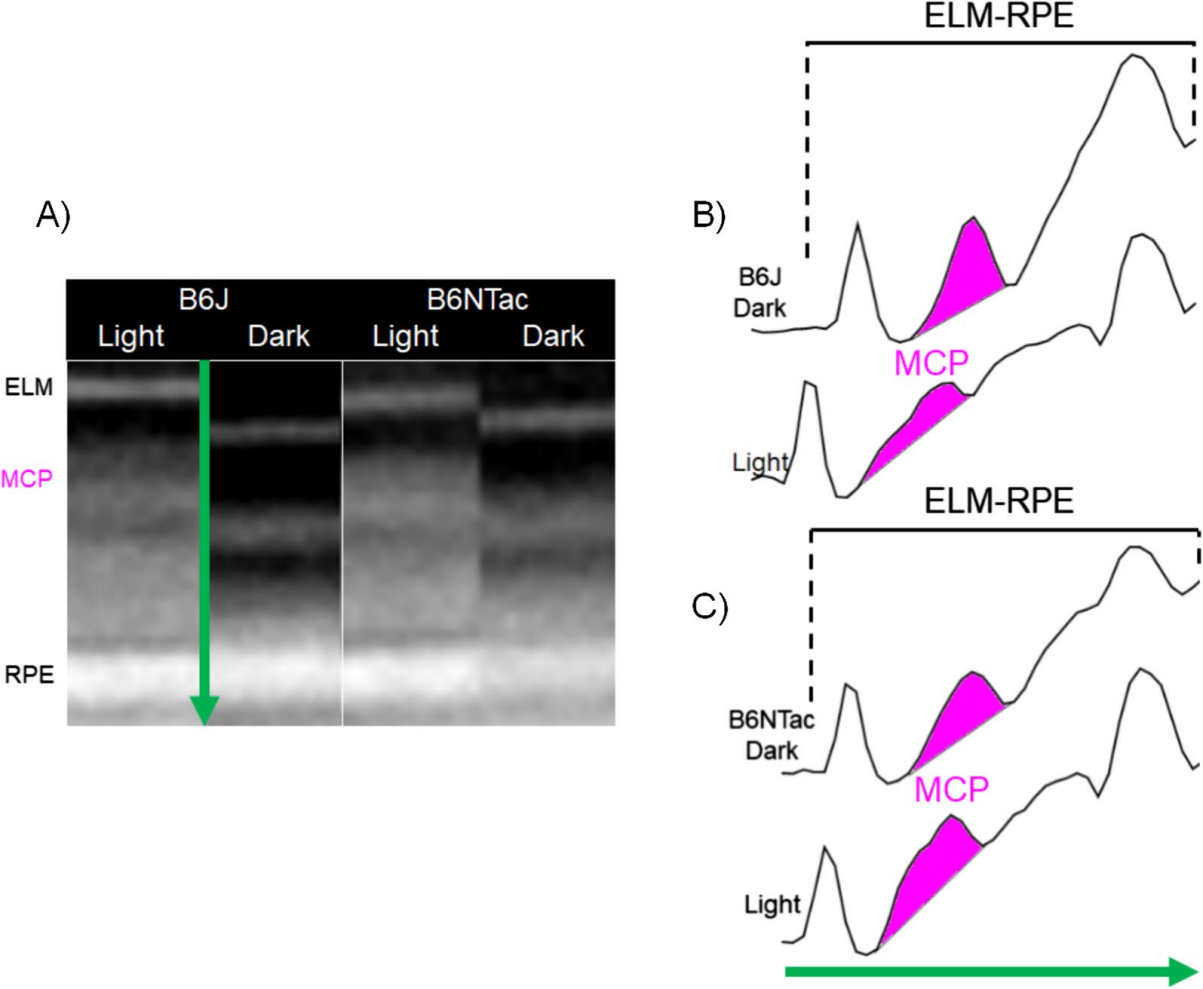
Summary of qualitative light–dark changes in WT substrains. **A** Representative OCTs of light and dark-adapted B6J and B6NTac mice outer retina. ELM, external limiting membrane; MCP/AR, region measured to evaluate the Mitochondria Configuration of Photoreceptors (MCP; also refered to as the ellipsoid zone); RPE, retinal pigment epithelium. Representative reflectivity profiles in the direction indcated by the green arrow, in inferior retina of **B** B6J and **C** B6NTac mice. ELM-RPE region is indicated by the horizontal line, MCP/AR is shown in pink. All reflectivity profiles are scaled the same; no y-axis is shown because units are arbitrary. No signal intensity normalization was performed for these indices

**Fig. 3:**
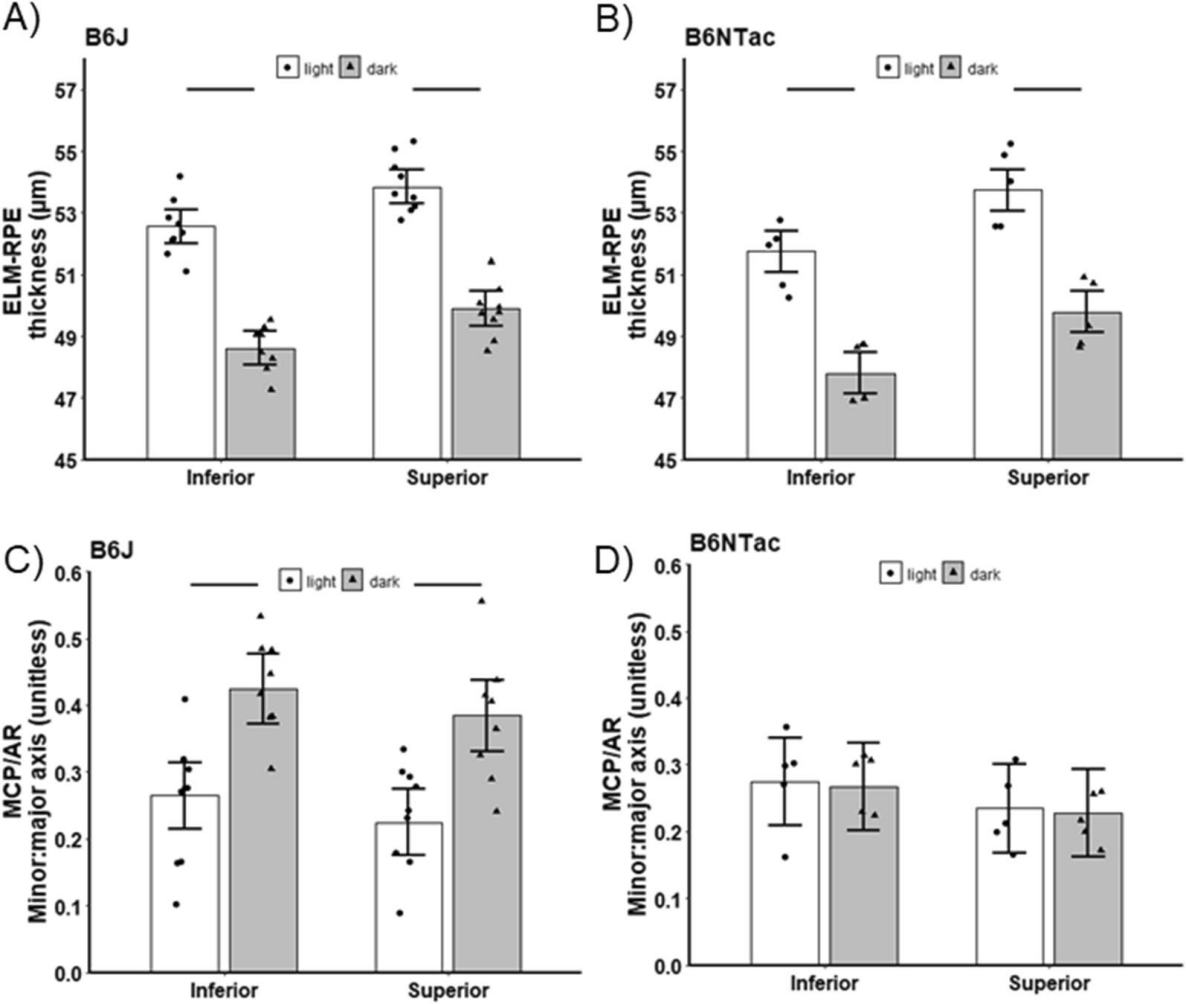
Summary of quantitative light–dark changes in bioenergy OCT biomarkers within B6J (**A**, **C**; n = 9 light, 9 dark) and B6NTac (**B**, **D**; n = 5 light, 5 dark) substrains. Black horizontal line indicates *P* < 0.05 (two-tailed, linear mixed-model analysis; mean ± 95% confidence interval [CI]). Individual data points represent the measured value for each mouse

Compared with light-adapted conditions, dark-adapted B6J and B6NTac mice showed a similar contraction of the ELM-RPE, consistent with the expected higher rod respiratory rate in the dark vs. light (B6J mean contraction = 3.98 µm, 95% CI 3.15–4.82 µm; B6NTac mean contraction = 3.88 µm, 95% CI 2.76–5.00 µm; contraction difference *p* = 0.87, Figs. 2 and 3). On the other hand, MCP/AR in B6J mice was greater in the dark than in the light (consistent with the presence of inefficient mitochondria [56]) (mean dark–light difference = 0.16, 95% CI 0.09–0.23, *p* = 0.0001; Figs. 2 and 3). However, MCP/AR was similar in the dark and light in B6NTac mice (consistent with the presence of efficient mitochondria [56]) (mean dark–light difference = −0.01, 95% CI −0.1 to 0.09, *p* = 0.87; Figs. 2 and 3). In summary, the lack of a light–dark change in B6NTac MCP/AR, unlike in B6J mice, is consistent with relatively greater mitochondrial efficacy.

### WT vs. 5xFAD substrains

Next, we evaluated our outcome metrics in 5xFAD mice for the two substrains.

#### OKT

**Fig. 4:**
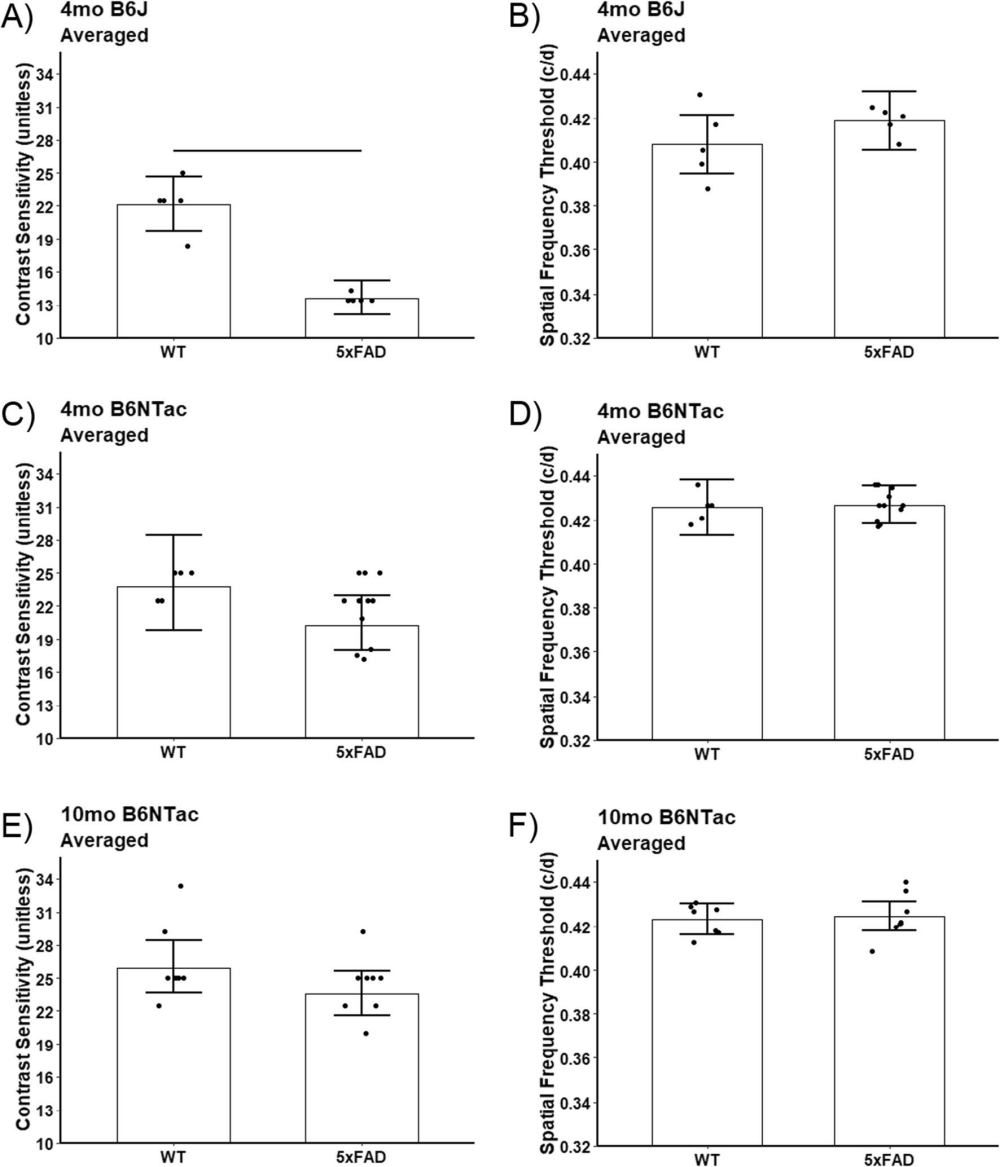
Summary of visual performance in controls and 5xFAD substrains. Impaired **A**) contrast sensitivity but not **B**) acuity was noted in 5xFAD B6J mice (WT, n = 5; 5xFAD, n = 5); 5xFAD B6NTac mice visual indices were unremarkable (**C**, **D**; 4 mo, middle row [WT, n = 5; 5xFAD, n = 11]; 10 mo, bottom row [**E**, **F**; WT, n = 7; 5xFAD, n = 7]). *Black horizontal line* indicates *P* < 0.05 (two-tailed, linear mixed-model analysis; mean ± 95% CI); *n* = 5 WT, *n* = 5 5xFAD. Individual data points represent the measured value for each mouse. Potential strain differences in both indices did not depend on which direction the grating moved, and means did not differ between directions so averages over both directions are presented

In 5xFAD B6J mice, CS was significantly lower-than-normal (0.6 fold change, 95% CI 0.5–0.7, *p* < 0.0001) whilst acuity did not achieve a statistically significant difference at 4 months (mean decrease = 0.01 c/d, 95% CI −0.01 to 0.03, *p* = 0.24, Fig. 4). 5xFAD B6NTac mice did not show evidence for reduced CS or acuity at 4 mo or 10 mo of age (*p* > 0.13, Fig. 4). The visibly apparent CS decline in 5xFAD B6NTac mice appears consistent, but smaller, than that in B6J mice, suggesting CS impairment may be present at this time point even if not statistically significant. These results raise the possibility that the trajectory of the visual performance declines in 5xFAD mice was a function of the presence of *Nnt*.

#### OCT energy biomarkers

**Fig. 5:**
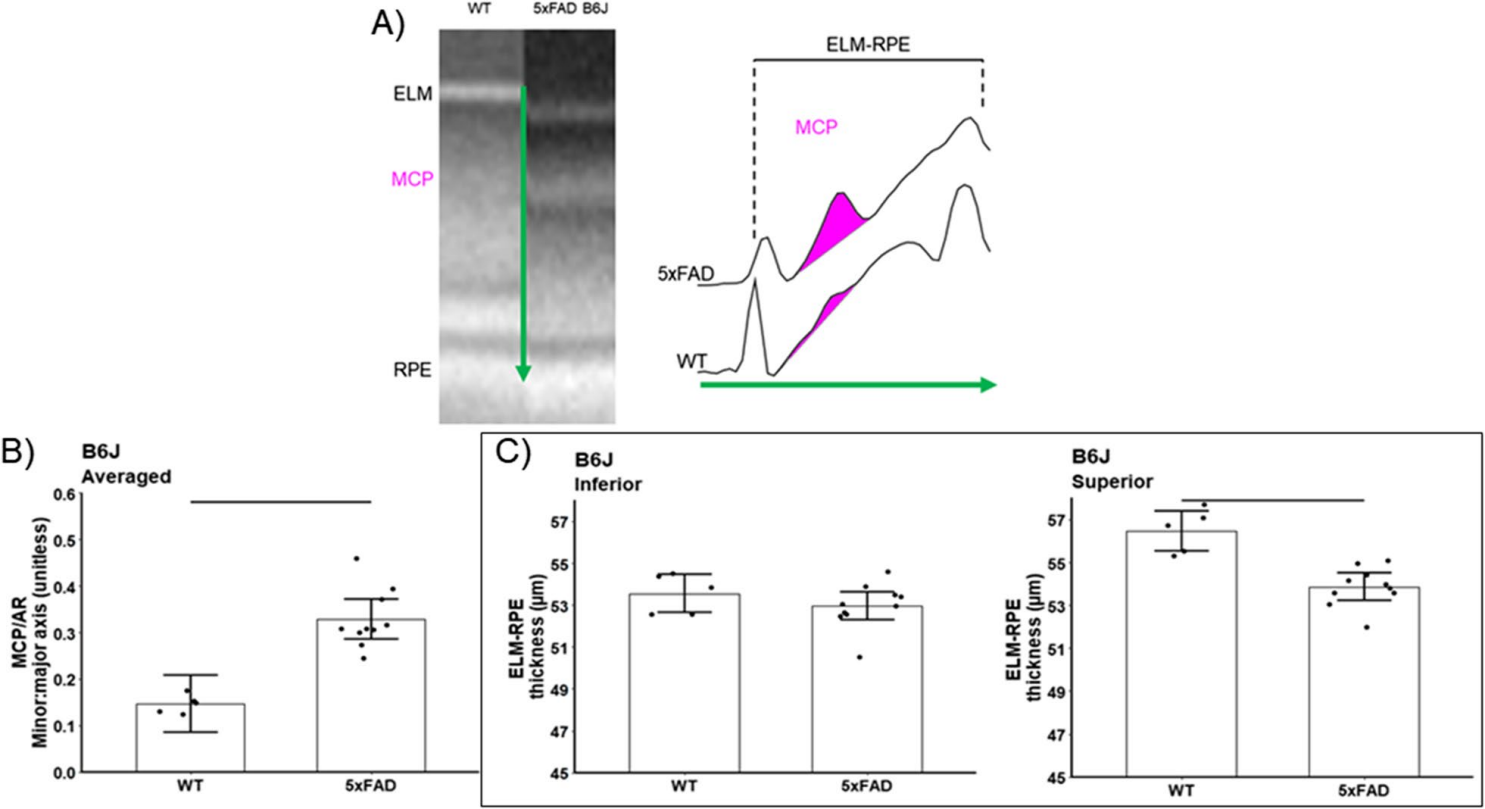
Summary of changes in WT (n = 5) vs. 5xFAD (n = 10) B6J mice. **A** Representative OCTs of light-adapted B6J mice in superior outer retina (left panel). Representative reflectivity profiles in the direction indicated by the green arrow, in superior retina (right panel). ELM-RPE region is indicated by the horizontal line, MCP/AR is shown in pink. **B** MCP/AR increased in 5xFAD mice suggesting inefficient mitochondria. Values did not depend on side, and means did not differ between sides so average over side are presented. **C** ELM-RPE contracted in 5xFAD mice suggesting hyperactivity

**Fig. 6:**
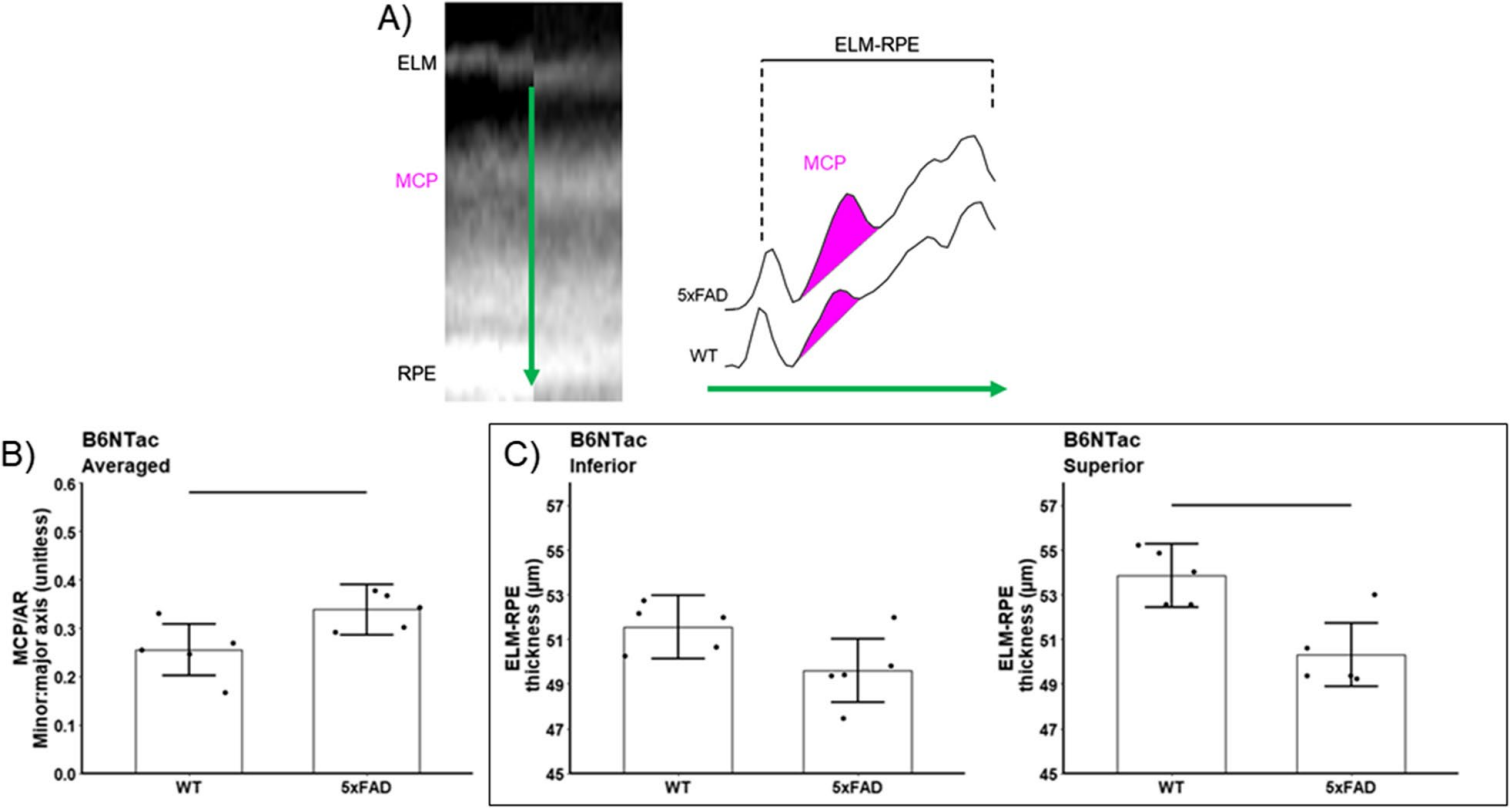
Summary of changes in WT (n = 5) vs. 5xFAD (n = 10) B6NTac mice. **A** Representative OCTs of light-adapted B6NTac mice in superior outer retina (left panel). Representative reflectivity profiles in the direction indicated by the green arrow, in superior retina (right panel). ELM-RPE region is indicated by the horizontal line, MCP/AR is shown in pink. **B** MCP/AR increased in 5xFAD mice suggesting inefficient mitochondria. Values did not depend on side, and means did not differ between sides so average over side are presented. **C** ELM-RPE contracted in 5xFAD mice suggesting hyperactivity

As shown in Figs. 5 and 6, both 5xFAD substrains showed contracted ELM-RPE in superior (B6J 5xFAD mean contraction = 2.61 µm, 95% CI 1.47–3.75 µm, *p* < 0.0001; B6NTac 5xFAD mean contraction = 3.54 µm, 95% CI 1.53–5.55 µm, *p* = 0.003), but not inferior (B6J 5xFAD mean contraction = 0.59 µm, 95% CI −0.54 to 1.73 µm, p = 0.29; B6NTac mean contraction = 1.95 µm, 95% I: −0.06–3.97 µm, *p* = 0.06) retina. These results are consistent with either hyperactive mitochondria or the presence of oxidative stress [38, 39, 56]. In addition, MCP/AR values in both 5xFAD substrains were supernormal in inferior and superior retina (B6J mean difference = 0.18, 95% CI 0.11–0.26, *p* < 0.0001; B6NTac mean difference = 0.08, 95% CI 0.01–0.16, *p* = 0.03; Figs. 5 and 6), in-line with highly active but inefficient mitochondria and / or oxidative stress [38, 39, 56, 83].

#### Retina laminar thickness

**Fig. 7:**
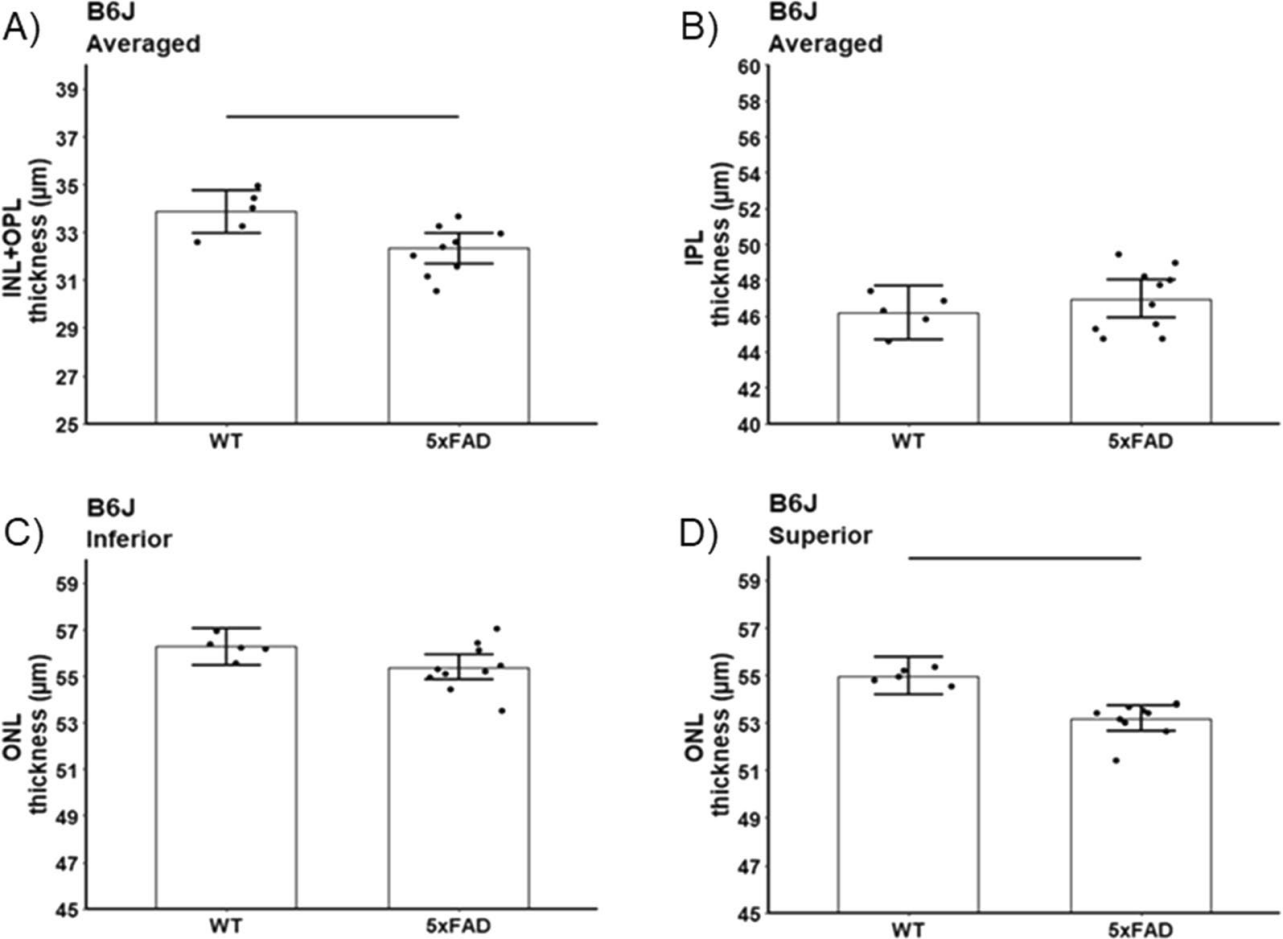
Summary of thicknesses in B6J WT (n = 5) vs. 5xFAD (n = 9) mice for these retinal layers: **A** inner nuclear layer + outer plexiform layer (INL + OPL), **B** inner plexiform layer (IPL), and **C** inferior and **D** superior outer nuclear layer (ONL). INL/OPL and IPL values did not depend on side, and means did not differ between sides so average over side are presented

**Fig. 8:**
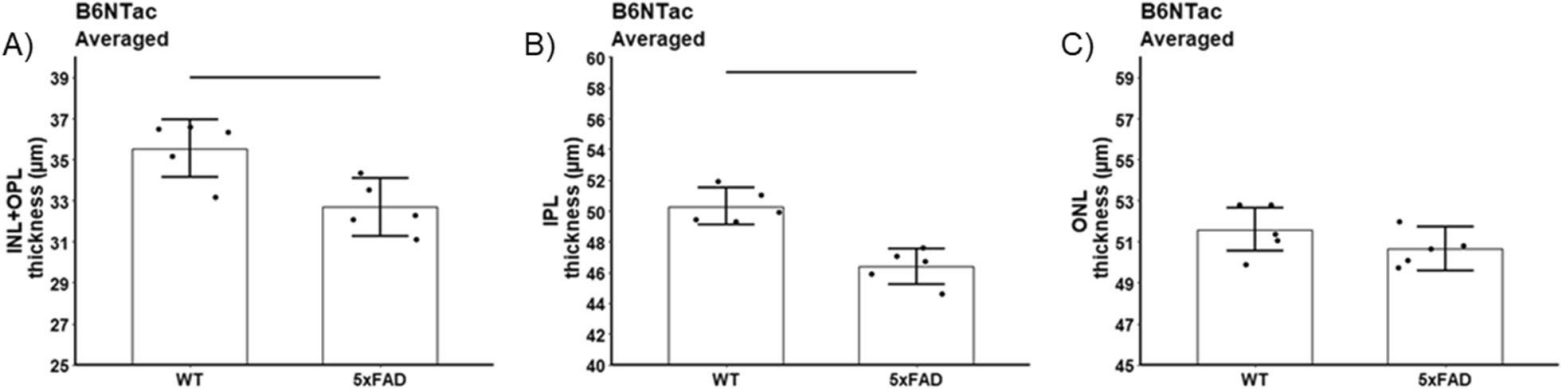
Summary of thicknesses in B6NTac WT (n = 5) vs. 5xFAD (n = 5) mice for these retinal layers: **A** inner nuclear layer + outer plexiform layer (INL + OPL), **B** inner plexiform layer (IPL), and **C** outer nuclear layer (ONL). INL/OPL, IPL, and ONL values did not depend on side, and means did not differ between sides so average over side are presented

Both 5xFAD substrains showed modest thinning of the INL + OPL layers (B6J 5xFAD mean thinning = 1.53 μm, 95% CI 0.43–2.64 μm; p = 0.01; B6NTac 5xFAD mean thinning = 2.86 μm, 95% CI 0.87–4.85 μm, *p* = 0.01; Figs. 7 and 8). 5xFAD B6J also showed reductions in superior ONL (mean thinning = 1.79 μm, 95% CI 0.84–2.74 μm, *p* = 0.001; inferior mean thinning = 0.88 μm, 95% CI −0.07 to 1.84 μm, *p* = 0.07); IPL was unremarkable (mean thinning averaged over side = −0.75 μm, 95% CI −2.60 to 1.09 μm; *p* = 0.40). In contrast, 5xFAD B6NTac mice showed thinning for the IPL (mean thinning averaged over side = 3.92 μm, 95% CI 2.24–5.60 μm, *p* = 0.001) but not ONL (mean thinning averaged over side = 0.93 μm, 95% CI −0.57 to 2.43 μm, *p* = 0.21; see Discussion).

### Treating 5xFAD B6J visual and OCT biomarker evidence for pathophysiology

Having shown both visual performance deficient and altered OCT energy biomarkers in B6J mice, we examined whether these indices in this substrain could be corrected pharmaceutically.

#### OKT

**Fig. 9:**
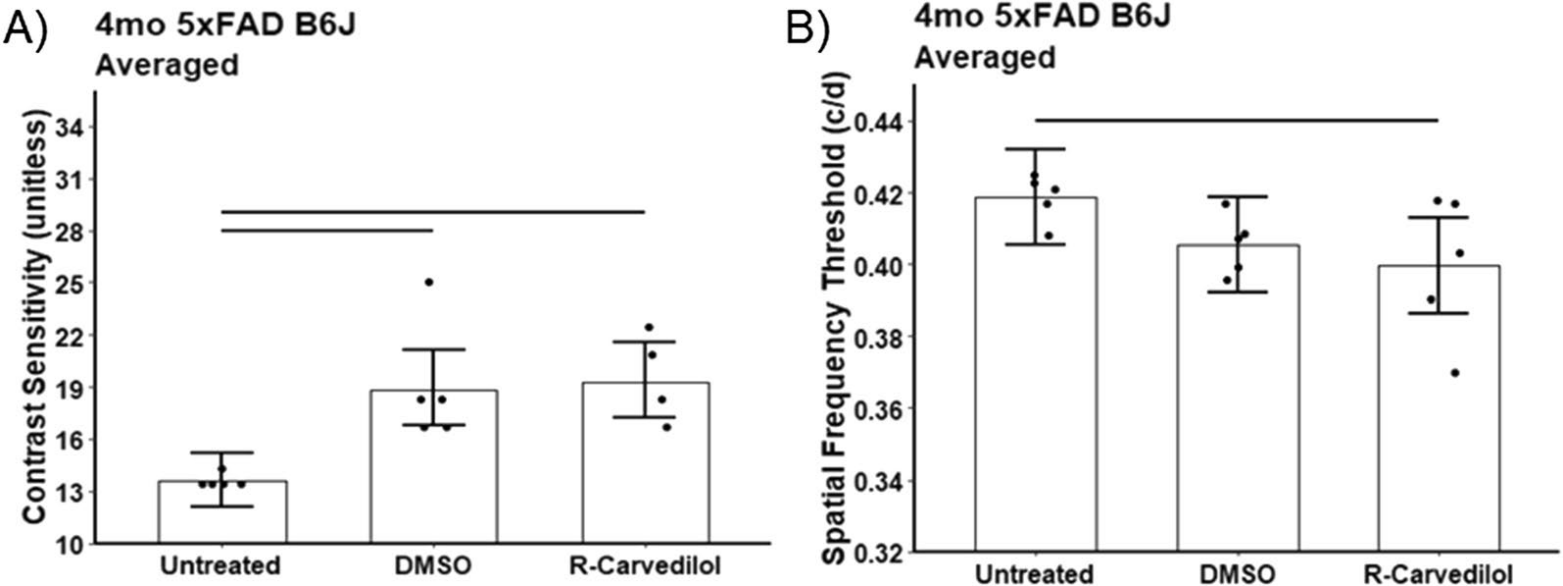
Summary of **A** contrast sensitivity and **B** acuity in 5xFAD B6J mice that were either untreated (n = 5) or treated with DMSO vehicle (n = 5) or R-carvedilol (n = 5). *Black horizontal line* indicates *P* < 0.05 (two-tailed, linear mixed-model analysis; mean ± 95% CI). Individual data points represent the measured value for each mouse. Potential strain differences in both indices did not depend on which direction the grating moved, and means did not differ between directions so averages over both directions are presented

Both DMSO alone and R-carvedilol + DMSO mitigated impaired contrast sensitivity in the untreated 5xFAD B6J mice (DMSO mean fold change: 1.4, 95% CI 1.2–1.6; *p* = 0.0005; R-carvedilol mean fold change: 1.4, 95% CI 1.2–1.7, *p* = 0.0003); R-carvedilol, but not DMSO, reduced acuity (DMSO mean change = −0.01 c/d, 95% CI −0.03 to 0.01 c/d, *p* = 0.15; R-carvedilol mean change = −0.02 c/d, 95% CI −0.04 to −0.0001 c/d, *p* = 0.05; Fig. 9). Based on these findings, DMSO appears to be the primary bioactive agent correcting visual performance declines in 5xFAD B6J mice.

#### OCT energy biomarkers

**Fig. 10:**
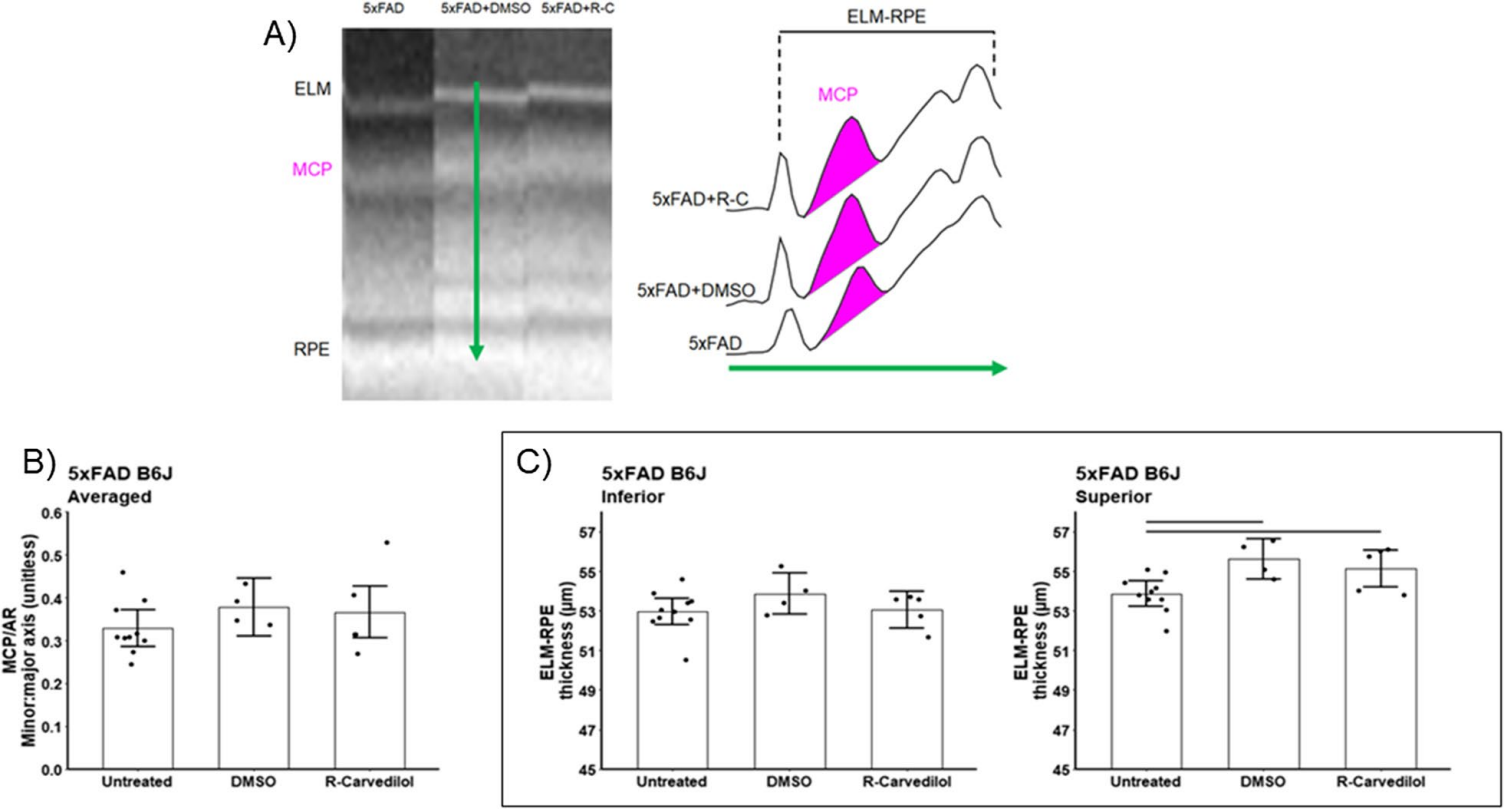
Summary of the impact of treatment on OCT energy-linked biomarker. **A** Representative OCTs from superior outer retina (left). ELM, external limiting membrane; MCP, region measured to evaluate the Mitochondria Configuration of Photoreceptors; RPE, retinal pigment epithelium. Representative reflectivity profiles in the direction indcated by the green arrow, in superior retina (right). ELM-RPE region is indicated by the horizontal line, MCP/AR is shown in pink. All reflectivity profiles are scaled the same; no y-axis is shown because units are arbitrary. No signal intensity normalization was performed for these indices. MCP/AR values did not depend on side, and means did not differ between sides so average over side are presented. **B** and **C** Summary of quantitative light–dark changes in bioenergy OCT biomarkers in untreated (n = 9), DMSO treated (n = 4), and R-carvedilol (R–C; n = 5) 5xFAD B6J mice. Black horizontal line indicates *P* < 0.05 (two-tailed, linear mixed-model analysis; mean ± 95% confidence interval [CI]). Individual data points represent the measured value for each mouse

Both DMSO alone and R-carvedilol + DMSO corrected the contraction of ELM-RPE in the superior retina of 5xFAD B6J mice (DMSO mean change = 1.76 μm, 95% CI 0.53–2.99 μm, *p* = 0.007; R-carvedilol mean change = 1.28 μm, 95% CI 0.15–2.42 μm, *p* = 0.03); inferior retina remained unremarkable (DMSO mean change = 0.89 μm, 95% CI −0.34 to 2.12 μm, *p* = 0.15; R-carvedilol mean change = 0.09 μm, 95% CI −1.05 to 1.22 μm, p = 0.87; Fig. 9). These results indicate that DMSO is also a bioactive agent that can correct the ELM-RPE pathophysiology in 5xFAD mice. On the other hand, neither DMSO nor R-carvedilol + DMSO modified MCP/AR (*p* > 0.22, Fig. 10). Possible reasons for this are presented in the Discussion.

#### Retina laminar thickness

**Fig. 11:**
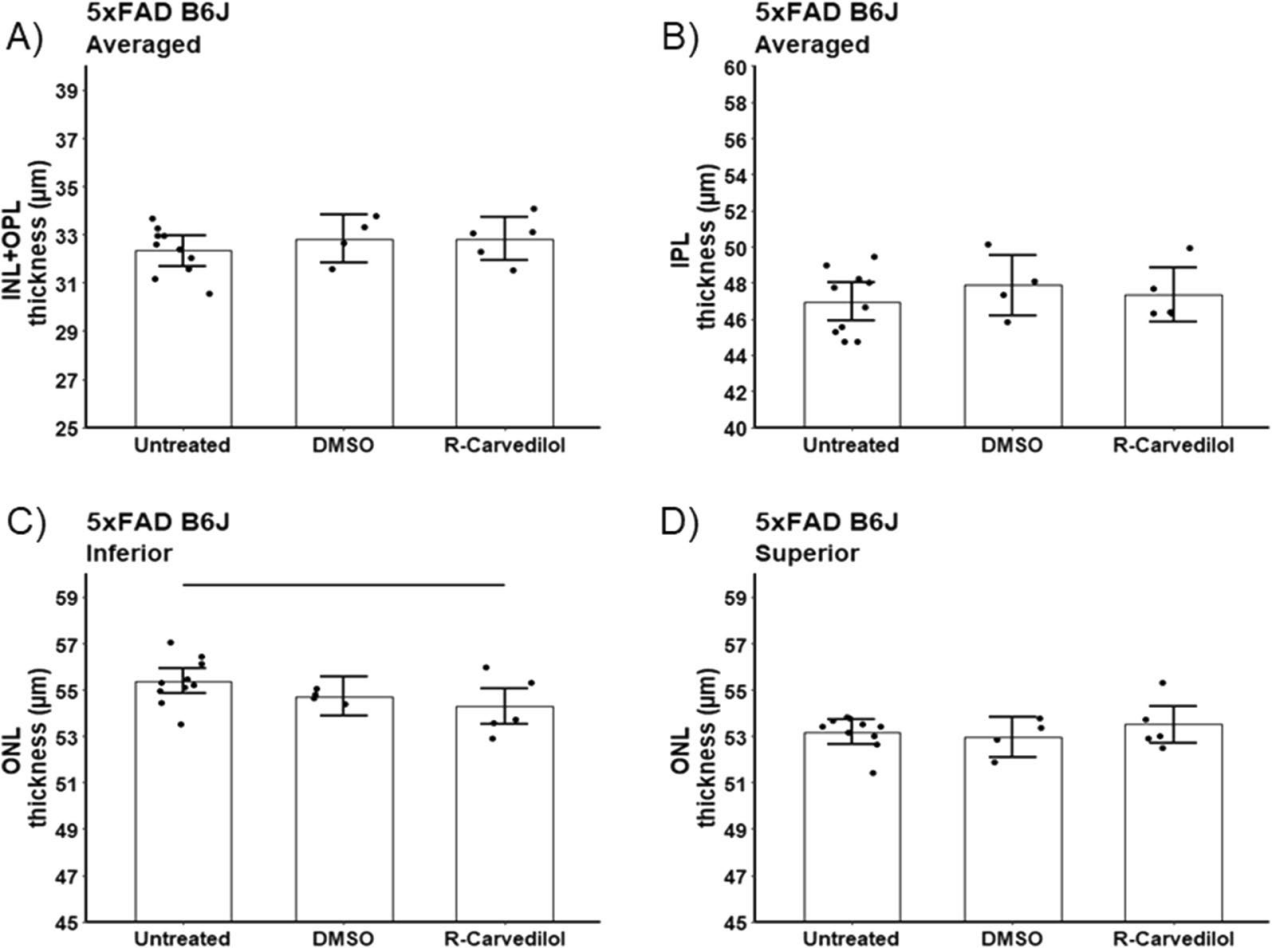
Summary of thicknesses in B6J that were either untreated (n = 9), DMSO treated (n = 4), and R-carvedilol (R–C; n = 5) mice for these retinal layers: **A** inner nuclear layer + outer plexiform layer (INL + OPL), **B** inner plexiform layer (IPL), and **C** inferior and **D** superior outer nuclear layer (ONL). INL/OPL and IPL values did not depend on side, and means did not differ between sides so average over side are presented

Neither DMSO nor R-carvedilol + DMSO corrected the modest thinning in 5xFAD B6J mice (Fig. 11) (see Discussion).

## Discussion

WT B6NTac mice show similar contrast sensitivity to WT B6J mice but have higher acuity and more efficient rod mitochondria as measured by OCT biomarkers: In this study, we probed the impact of *Nnt* on visual performance and rod respiratory OCT biomarkers. Little evidence was found that CS is driven by photoreceptor mitochondria efficacy given the similarity between WT B6NTac and B6J mice [84]. It is not clear if the small but relatively higher acuity in WT B6NTac mice over that in WT B6J mice is biologically meaningful. Mice on a B6J background harbor a truncated, non-functional *Nnt* that leads to a reduction in the efficacy of ATP synthesized [47]. In contrast, B6NTac mice express *Nnt* and so presumably contain normal levels of ATP [47]. Contraction of the ELM-RPE is a proxy for acidification of the subretinal space [39–46, 85, 86]. For example, during dark-adaptation, rod cGMP accumulates in the outer segment and maintains a high energy demand to keep cyclic nucleotide-gated channels persistently open, an event that depolarizes the rod membrane and increases ion pumping / mitochondrial energy utilization for dark adaptation compared to light-adaptation [39–45]. Higher mitochondrial activity is associated with increased levels of lactate, CO_2_ and waste water, resulting in an acidified subretinal space that triggers an increase in apical RPE co-transporter-based water removal with concomitant shrinkage of the ELM-RPE region [40, 46, 85, 86]. In addition, if the subretinal space becomes acidified by, for example, oxidative stress or the carbonic anhydrase inhibitor acetazolamide during low energy demand conditions like light adaptation, the ELM-RPE contracts [38, 43, 45, 51, 52, 85–89]. Both WT B6J and B6NTac mice in this study show the expected light–dark change in ELM-RPE.

Current evidence suggests that MCP/AR reflects the mitochondria distribution and number within the photoreceptor ellipsoid, and as such is a promising non-destructive index of photoreceptor mitochondria efficacy [90, 91]. For example, B6J mice show a dark > light MCP/AR pattern, whilst 129S6/ev mice show a dark = light MCP/AR pattern [50–52, 56, 58]. In the present study, B6NTac mice also show a dark = light MCP/AR pattern. Considered together with the fact that B6NTac mice show evidence for high energy demand acidification of the subretinal space (i.e., ELM-RPE contraction), rod mitochondria in B6NTac mice thus appear to be more efficient than in B6J mice.

### CS impairment in 5xFAD B6J mice

CS is reduced in patients early in the course of AD, a morbidity that promotes falling and reduced survival [13–23]. Here, we showed reduced CS (without evidence for visual acuity deficits) in 4 mo 5xFAD B6J but not a significant difference in 4 or 10 mo 5xFAD B6NTac mice [10–12]. However, the observed CS declines in 10 mo WT and 5xFAD B6NTac are consistent with, but smaller than, those in B6J raising the possibility that CS decline starts later than in B6J mice. A previous report suggested that 5xFAD B6NTac developed an amyloidogenic phenotype and a later onset of cognitive dysfunction at a slower rate than 5xFAD B6J mice; head-to-head comparisons are still needed however [12]. It remains to be determined if a delay in CS impairment in 5xFAD B6NTac mice compared to 5xFAD B6J mice occurs and is a consequence of a relatively higher photoreceptor mitochondria efficacy in the B6NTac mice.

### OKT evidence for oxidative stress in 5xFAD B6J mice

Since CS was affected early in 5xFAD B6J mice, we used this model to test for treatment benefits. The impaired CS in 4 mo 5xFAD B6J mice could be corrected by R-carvedilol. R-carvedilol is an enantiomer that limits the open-time of the endoplasmic reticulum (ER) ryanodine receptor type 2 (RyR2) calcium channel, likely has anti-oxidant properties like the racemic mixture, and corrects cognitive performance decline and neuronal hyperactivity in experimental models that are genetically engineered to mimic hallmark AD neuropathologies [7, 26, 59–63, 65, 67, 68, 72–74]. Earlier studies investigated the racemic mixture of S- and R- carvedilol enantiomers in experimental retinal degenerative models, and in patients with AD in a phase 4 study (NCT01354444) [69, 69, 92–94]. However, S- and R- carvedilol enantiomers have different mechanisms-of-action and it is unclear whether the R- enantiomer of carvedilol shows the same benefits as the racemic mixture [62, 69].

Somewhat surprisingly, the reduction in CS in 5xFAD B6J mice could be corrected by administering a very low dose of DMSO (0.01%), the vehicle used for bringing R-carvedilol into solution [62, 72]. Commonly thought to be a dose that is largely without biologic activity, 0.01% DMSO is routinely used as a vehicle, even though several studies have shown that very low doses of DMSO can stimulate respiration and excitability in brain slices and even increase cellular calcium ion content from intracellular stores in primary cultures [95–100]. Further, DMSO treatment has been reported as a neuroprotective agent in the retina and visual system in clinical and experimental studies [100–104].

We note that DMSO is well-studied as a scavenger of hydroxyl free radicals that can contribute to an oxidative stress environment [101, 102]. Retinal oxidative stress per se has been associated with a reduced CS but not acuity in some studies based on its response to anti-oxidants [82, 105]. The above considerations show that DMSO alone, a compound with anti-oxidant properties, corrected CS impairment and support the notion of oxidative stress in the retina of 5xFAD B6J mice. The OCT results (discussed below) further show that DMSO corrects an abnormally contracted ELM-RPE in 5xFAD B6J mice, and thus is a likely demonstration for the presence of oxidative stress (i.e., QUEST OCT); further studies are clearly warrented [38].

### 5xFAD mutations in B6NTac mice lower mitochondria efficacy based on OCT biomarkers

Compared to WT light adapted B6NTac mice, 5xFAD B6NTac mice have a contracted ELM-RPE in the superior retina; a result that mirrors that observed in 5xFAD B6J mice [24]. The major driver of ELM-RPE contraction is low subretinal pH (that can occur, for example, with oxidative stress) [24, 38, 40, 45, 50, 52, 57, 58, 105]. We note that before the appearance of AD pathology, neuronal acidification as a consequence of producing ATP through glycolysis (Pasteur effect) and / or the presence of inadequate suppression of an excessive production of free radicals (i.e., oxidative stress) has been reported [106–110]. Such considerations raise the possibility for outer retinal oxidative stress in 5xFAD mice since the hydroxyl free radical scavenger DMSO corrected the contracted ELM-RPE (see below). More work is needed to explain why the inferior retina of 5xFAD mice did not show a significantly contracted ELM-RPE.

As noted above, dark adapted WT B6NTac mice present with the expected contracted ELM-RPE and no change in MCP/AR from light adapted conditions, consistent with efficient rod mitochondria [56]. However, light adapted 5xFAD B6NTac mice show an elevated MCP/AR compared to dark-adapted WT B6NTac mice, a pattern that resembles less efficient rod mitochondria seen in light-adapted 5xFAD B6J mice compared to WT B6J mice [24]. In support of the above considerations, we note that inducing acidification with acetazolamide in light-adapted WT B6J mice (inefficient mitochondria) resulted in MCP/AR increasing whilst in S6 mice (efficient mitochondria) MCP/AR decreased (in press). Taken together, an increased MCP/AR, in the presence of contracted ELM-RPE (implying acidification of the subretinal space), is in-line with how inefficient rod mitochondria respond to acidification in the 5xFAD mice. Since acidification can arise from a variety of conditions, such as oxidative stress, these results alone are insufficient to identify a particular factor contributing to the mitochondria dysfunction we showed in the rods of 5xFAD B6NTac mice.

### OCT evidence for rod oxidative stress in 5xFAD B6J mice

Previously, we reported that anti-oxidant correction of a contracted ELM-RPE indicates the presence of oxidative stress, a likely consequence of subretinal space acidification [111, 112]. As noted above, DMSO has anti-oxidant properties [95–102, 113–115]. Thus, correcting ELM-RPE contraction following DMSO suggests a protective effect against early 5xFAD-induced declines in mitochondria abnormalities (and CS, above) are linked to oxidative stress.

In contrast, MCP/AR remained supernormal after DMSO (and R-carvedilol) treatment. We considered two possible explanations for these results. First, MCP/AR unresponsiveness to DMSO (or R-carvedilol) could indicate that irreversible oxidative damage occurred to the rod mitochondria produced by oxidative stress before treatment was started in this study. Second, mitochondria dysfunction might arise from factors other than oxidative stress / oxidative damage and thus would be unchanged by DMSO or R-carvedilol treatment. One possible alternative dysfunction is an early increase in the level of mitochondrial fission protein, DRP1, as reported in patients and APP/PS1 mice [116–118]. It has been suggested that mitochondria are reflective sources, and thus increased mitochondrial fragmentation could increase MCP/AR as measured by OCT [119, 120]. To begin to investigate these hypotheses, studies that administer DMSO treatment earlier in male and female 5xFAD mice are needed.

#### Retinal laminar anatomy in 5xFAD mice

In this study, B6J and B6NTac substrains both demonstrated modest thinning of the INL + OPL, distinct patterns for thinning of other retinal layers, and atrophy that was unresponsive to treatment. Other studies find that 5xFAD mice show no change or thicker or thinner retina layers compared to controls that might arise from lab-to-lab variations (e.g., fixation (histology) versus no fixation (OCT), age differences, and/or bias in manual segmentation or lack of side comparisons in OCT studies) [121–123]. More work is needed to identify what is responsible for laminar thinning in the 5xFAD mice in order to understand why neither DMSO nor R-carvedilol was beneficial in this study [123].

#### Mouse model considerations

We appreciate that experimental mouse models like the 5xFAD mouse do not fully capture all aspects of AD disease seen clinically [2]. Nonetheless, features of AD can be modelled in experimental mouse models and used for hypothesis testing and discovery not possible in patients [2]. Identifying changes in imaging biomarkers with treatment, and probing mechanisms-of-action in, for example, the 5xFAD mouse can raise new treatment possibilities and highlight limitations in the development of clinical interventions, as we have shown herein. In this study, we tested how genetic factors can influence disease trajectory before the appearance of AD pathology in the retina as well as the evaluation of novel treatments like 0.01% DMSO.

It is not currently possible to perform widespread screening in patients for early neuronal mitochondrial dysfunction, oxidative stress, or oxidative damage because of the limited availability of expensive imaging tools like positron emission tomography or MRI, nor to apply conventional ex vivo assays to interrogate the in vivo condition [34, 124–129]. Thus, new low cost imaging approaches with translational energy biomarkers are needed, the problem we and others are approaching herein with OCT [24, 31–38].

## Summary

**Fig. 12:**
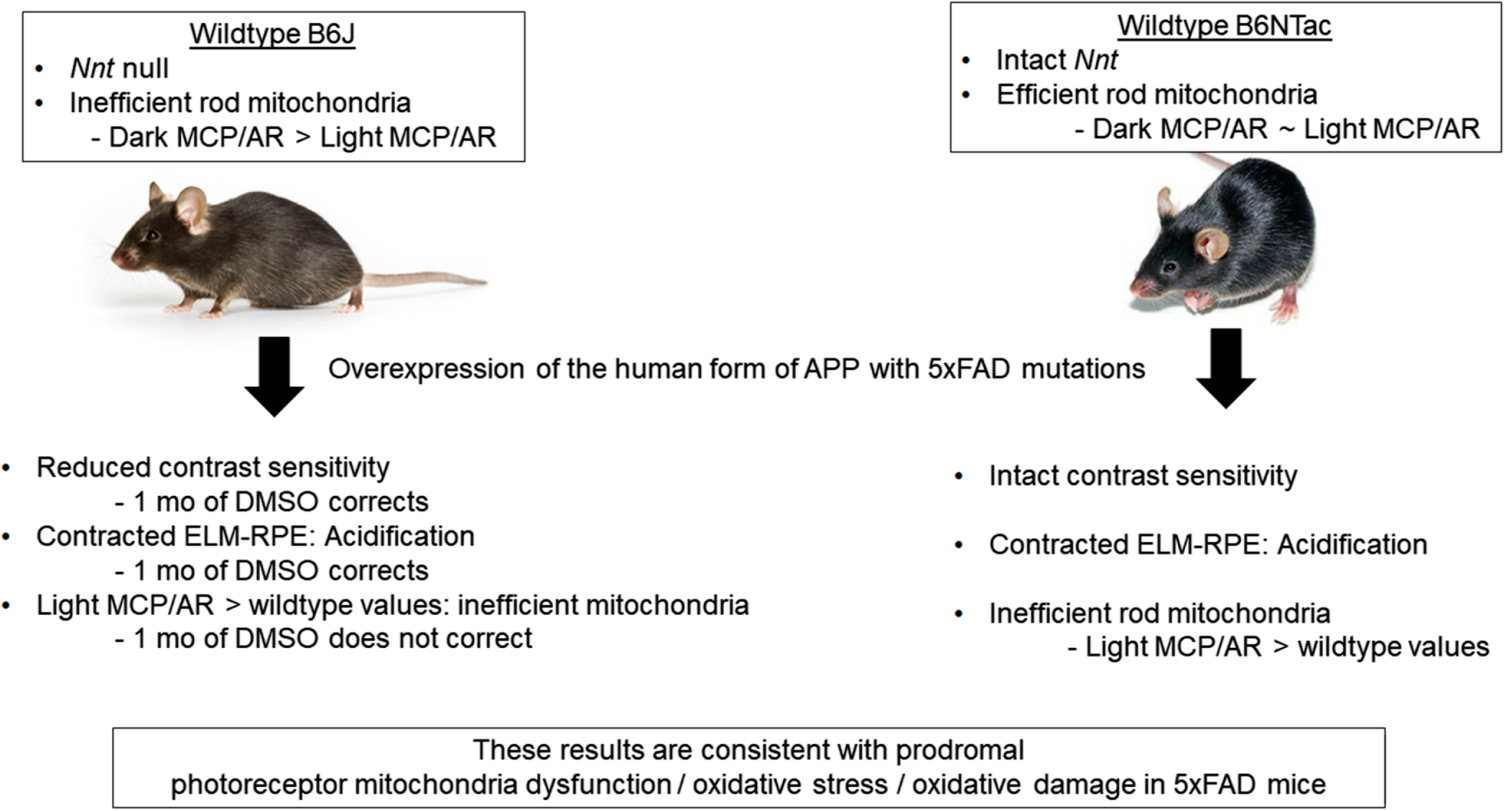
Summary of major findings and conclusion of this study

A summary of the major findings and conclusion are presented in Fig. 12. We found little evidence for a link between visual performance and rod mitochondrial efficacy OCT biomarkers. Our data supported the notion that B6NTac rod mitochondria were more efficient than in B6J mice at baseline but that B6NTac mice overexpressing the human form of APP with 5xFAD mutations show a reduction in mitochondria efficacy. These mice also showed acidification of the subretinal space in both substrains based on the contracted ELM-RPE and increased MCP/AR. The present results neither support nor rule out rod hyperactivity early in 5xFAD mice [24]. However, given the established crosstalk between mitochondria abnormalities and oxidative stress, and the evidence presented herein including that the hydroxyl free radical scavenger DMSO corrected both CS and ELM-RPE contraction, but not MCP/AR changes in 5xFAD B6J mice, we speculate that a combination of photoreceptor mitochondria dysfunction / oxidative stress / oxidative damage occurs in 5xFAD mice regardless of the presence of *Nnt*, a rare mutation in humans that leads to familial glucocorticoid deficiency, adrenal insufficiency and hypothyroidism [35, 130–135].

The results of the present study further support the novel use of OCT photoreceptor energy biomarkers as an accessible and cost-effective approach for early diagnosis and evaluation of treatment efficacy in animal AD models [24, 31–38]. Because conventional imaging biomarkers are expensive and have limited availability for widespread evaluation of early neuronal mitochondrial dysfunction and / or oxidative stress in AD, and because OCT is a routine procedure world-wide, we anticipate that clinical application of the present biomarkers to be important in the early diagnosis and treatment of patients with mild cognitive impairment [34, 124–129].

## Data Availability

The datasets during and/or analyzed during the current study available from the corresponding author on reasonable request.

## Abbreviations

ACZ: Acetazolamide
AD: Alzheimer’s Disease
B6J: C57BL/6J mice (Jackson Laboratory)
B6NTac: C57BL/6NTac mice (Taconic Laboratory)
cGMP: Cyclic guanosine monophosphate
CS: Contrast sensitivity
DMSO: Dimethyl sulfoxide
DRP1: A mitochondrial fission protein
ELM-RPE: The distance/thickness between the external limiting membrane and the retinal pigment epithelium of the retina
INL: Inner nuclear layer
MCP/AR: Mitochondrial configuration of photoreceptors measured by its aspect ratio
MB: Methylene blue
NADH: Nicotinamide adenine dinucleotide
NADPH: Nicotinamide adenine dinucleotide phosphate
Nnt: Nicotinamide nucleotide transhydrogenase
OCT: Optical coherence tomography
OKT: Optokinetic tracking
ONL: Outer nuclear layer
OPL: Outer plexiform layer
